# A Hybrid Machine Learning Framework to Improve Morphological Trait Recovery in Avian Datasets

**DOI:** 10.1002/ece3.73173

**Published:** 2026-03-24

**Authors:** Yu Bai, Pengfei Song, Shimin Wen, Hongjie Zhu, Yuang Wang, Huazhang Wang, Xixi Feng, Bo Ma, Daji Ergu, Fangyao Liu

**Affiliations:** ^1^ College of Computer Science and Artificial Intelligence Southwest Minzu University Chengdu China; ^2^ Sichuan Zoige Alpine Wetland Ecosystem National Observation and Research Station Southwest Minzu University Chengdu China; ^3^ Qinghai Provincial Key Laboratory of Animal Ecological Genomics, Northwest Institute of Plateau Biology Chinese Academy of Sciences Xining China; ^4^ Key Laboratory of Electronic Information Engineering Southwest Minzu University Chengdu China

**Keywords:** biodiversity informatics, hybrid machine learning, missing data imputation, morphological traits, radial basis function networks

## Abstract

Missing data in morphological trait datasets pose a persistent challenge to ecological and evolutionary research, frequently compromising model inference and predictive accuracy. We propose THORBFNN, a three‐stage hybrid imputation framework that integrates regularized K‐means clustering, Radial Basis Function Neural Networks (RBFNNs), and hierarchical Bayesian optimization to accurately recover missing avian morphological traits. The framework partitions species into clusters using regularized K‐means, enhancing the preservation of local morphological structure through inter‐cluster separation. Within each cluster, RBFNNs model nonlinear dependencies among traits using input features selected by Pearson correlation with the target trait. Key hyperparameters such as the number of clusters and RBF width are optimized via hierarchical Bayesian optimization to balance generalization and model complexity. When applied to a global avian trait dataset comprising over 10,000 individuals and 11 morphological traits, THORBFNN outperforms K‐nearest neighbors and Random Forest imputation across four focal traits, achieving higher *R* ^2^ and lower errors (THORBFNN: *R* ^2^ = 0.9003, RMSE = 0.1652, MAE = 0.1096; KNN: *R* ^2^ = 0.8864, RMSE = 0.1668, MAE = 0.1248; Random Forest: *R* ^2^ = 0.8573, RMSE = 0.2134, MAE = 0.1584). Ablation experiments comparing models trained on complete cases versus mean‐imputed data confirm that THORBFNN captures genuine trait covariation rather than statistical artifacts. THORBFNN requires no phylogenetic information and scales efficiently to datasets with thousands of individuals, offering a practical pathway for integrating machine learning into biodiversity trait analysis.

## Graphical abstract

Our hybrid models integrating local pattern recognition, nonlinear mapping, and adaptive optimization are anticipated to elevate the accuracy of the imputation while preserving the distinctiveness of biological datasets, thus providing a more reliable foundation for ecological and evolutionary research.

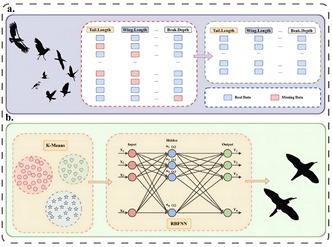

## 1 Introduction

Quantitative traits serve as a fundamental basis for biological research, with morphological traits playing a central role in ecological and evolutionary studies (Johnson et al. 2021). However, existing biological trait databases are often incomplete, and many species or traits contain missing measurements (García‐Laencina et al. 2010; Seyed Tabib et al. 2020). Previous work has shown that omitting species or traits can seriously distort estimates of community functional structure and evolutionary dynamics, thereby reducing predictive accuracy and introducing systematic biases (Poggiato et al. 2021). Therefore, it is essential to handle missing values in morphological trait data appropriately in order to retain as many samples as possible and avoid biased results (Debastiani et al. 2021). Effective missing‐value imputation is critical for ensuring the reliability and robustness of macroecological and evolutionary analyses. Traditional approaches include list‐wise deletion, in which individuals with missing entries are removed, or simple statistical substitutions such as mean or median filling. Although complete‐case analysis is straightforward to implement, it often discards a large number of valuable samples and may introduce bias (Bowler et al. 2025). Single‐value imputation methods fail to account for correlations among features and typically yield low accuracy (Mirzaei et al. 2022). Empirical studies have demonstrated that, although mean and median imputation preserve all available samples, they tend to distort the distribution of imputed values (Rizvi et al. 2023; Wang et al. 2023). In contrast, imputation based on traditional machine learning techniques generally achieves better performance (Little et al. 2024).

Additionally, multiple‐imputation methods can be effective under specific assumptions; however, their success strongly depends on the underlying missing‐data mechanism, and their performance deteriorates when data are missing not at random or exhibit strong bias (Enders 2022). Overall, conventional statistical imputation methods show clear limitations in generalization capability and in their ability to capture complex data structures.

To address these challenges, this paper proposes a hybrid imputation method that integrates clustering, neural networks, and statistical optimization techniques. The method proceeds in three stages:

- First, the morphological trait matrix is clustered using K‐means, grouping bird species with similar morphological features into multiple clusters to uncover local data structures.
- Second, a Radial Basis Function (RBF) neural network is constructed for each cluster to predict missing values within that group, enabling the modeling of complex nonlinear relationships.
- Third, feature correlation analysis is employed to select input features most relevant to the target variable, and Bayesian optimization is applied to automatically tune key hyperparameters, including the number of K‐means clusters and the initialization centers of the RBF network, improving convergence speed and mitigating overfitting risks.

In practice, the feature–target correlations are computed separately for each trait using only the observed entries, which helps to reduce multicollinearity and restrict the model to biologically informative predictors while avoiding circularity in the imputation process. By combining the global structure discovery capability of clustering with the nonlinear approximation power of RBF networks and the adaptive tuning provided by Bayesian optimization, the proposed framework aims to improve the accuracy and robustness of trait imputation while remaining computationally feasible for large avian datasets.

In this study, we evaluate the proposed method on a global avian morphological trait dataset containing more than 10,000 individuals and 11 key traits with heterogeneous missing‐value patterns. We compare the proposed framework with representative machine‐learning baselines, including K‐nearest neighbors and Random Forest, under different missingness proportions and mechanisms. Furthermore, we examine how the imputed values affect downstream ecological analyses, focusing on multivariate trait axes and functional diversity indices to illustrate the ecological relevance of the imputation results. By combining the global structure discovery capability.

## 2 Related Work

In the domain of missing value imputation, the integration of traditional statistical methods with contemporary machine learning techniques offers a wide range of solutions to improve data integrity (Chakraborty et al. 2017; Gond et al. 2021). Early univariate imputation strategies (e.g., mean/median substitution) have been progressively supplanted by approaches grounded in distribution modeling due to their neglect of variable inter‐dependencies and introduction of systematic biases (Newman 2014). Algorithms epitomized by K‐Nearest Neighbors (KNN) and Multiple Imputation by Chained Equations (MICE) have markedly enhanced imputation accuracy through mechanisms such as local similarity assessment or iterative multivariate regression (Awawdeh et al. 2022). However, these algorithms face challenges such as the curse of dimensionality and inadequate representation of nonlinear relationships (Adhikari et al. 2022; Lisboa et al. 2023). For example, KNN's distance metric can falter in high dimensional spaces, leading to imputed values that diverge from the true distribution, while MICE's reliance on specific assumptions about the missingness mechanism restricts its efficacy under nonrandom missing patterns (Liu et al. 2023). Recently, deep learning technologies have caused a paradigm shift in imputation methodologies. Generative Adversarial Networks (GANs) and Variational Autoencoders (VAEs) (Wabina et al. 2025; Tiwaskar et al. 2025), by implicitly learning underlying data distributions, excel in modeling intricate non‐linear relationships. However, these models are encumbered by significant data requirements and limited interpretability (Hu et al. 2024). Concurrently, ensemble learning frameworks, exemplified by Random Forest imputation, mitigate the bias inherent in single‐model approaches through aggregation of multiple model predictions, though they risk excessive smoothing which can obliterate unique feature characteristics (Innocent 2024). Research indicates that parameter adaptation strategies based on Bayesian optimization can substantially increase the generalizability of such models.

Hybrid imputation methods, which amalgamate the strengths of diverse technologies inferent stages, have emerged as a leading research area (Shah et al. 2017; Silvestro et al. 2024). Exemplary strategies include: (1) localized modeling predicated on clustering based pre‐segmentation, wherein neural networks are employed for region‐specific imputation following substructure identification via K‐means, thus alleviating issues related to global model overfitting; (2) multi‐model collaborative architectures, combining Support Vector Machines (SVM) with Radial Basis Function (RBF) networks to leverage SVM's classification boundary determination and RBF's nonlinear fitting capabilities, thereby bolstering imputation robustness; and (3) meta‐heuristic optimization‐driven parameter searches, like employing chaotic orthogonal learning to enhance the Slime Mold Algorithm, aimed at accelerating model convergence and evading local optima. While these methodologies have demonstrated success in areas such as traffic flow prediction and medical data analysis, their application to ecological morphological data remains largely unexplored.

Within biology, although techniques such as Bayesian Hierarchical Probabilistic Matrix Factorization (BHPMF) have been applied to the imputation of morphological traits in avian species, their heavy reliance on phylogenetic development priors limits their broad applicability (Little 2021; Ling and Jafarpour 2024). There is an urgent need for imputation frameworks capable of adapting to intrinsic data structures—such as spatial heterogeneity and evolutionary correlations—without dependency on external priors. Consequently, hybrid models integrating local pattern recognition, nonlinear mapping, and adaptive optimization are anticipated to elevate imputation accuracy while preserving the distinctiveness of biological datasets, thus furnishing a more dependable foundation for ecological and evolutionary inquiries.

## 3 Method and Data Analysis

### 3.1 Data Source and Acquisition

Datasets in this study, we utilize the Data S1 from the AVONET database (AVONET n.d.), which provides repeated morphological measurements for a subset of individual birds sampled across 181 countries. The data set includes 11 key morphological traits, such as beak length, beak width, wing length, tail length, and handwing index, that are functionally important in avian ecology. Several traits exhibit substantial missingness; for example, Beak.Length Nares is missing in more than 50% of entries, and Kipps.Distance in over 35%, reflecting the typical incompleteness of ecological datasets of the real world. This dataset offers distinct advantages for missing value imputation. First, the presence of repeated measurements for the same specimen enables the estimation of measurement error and enhances the robustness of the model to observational noise. Second, the high dimensionality of the trait data provides a suitable setting for evaluating imputation performance in complex, continuous‐feature spaces. Third, its broad taxonomic and geographic coverage ensures strong ecological representativeness, supporting the development of imputation models with improved generalizability.

### 3.2 Problem Setting and Data Preprocessing

**FIGURE 1:**
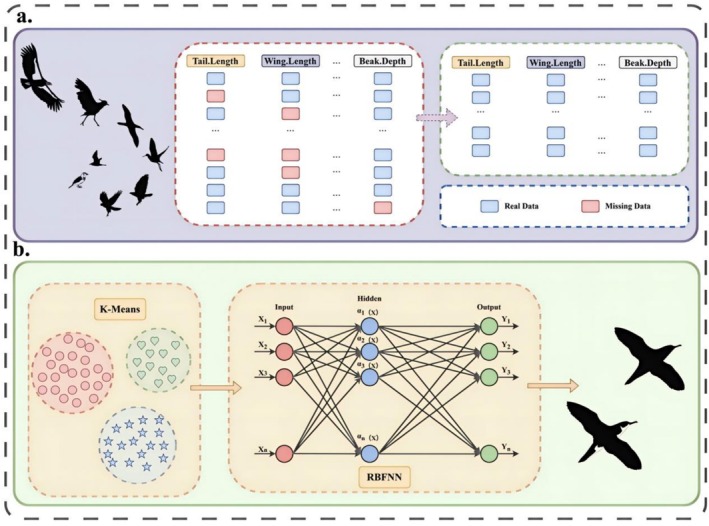
The architecture of the proposed Three‐stage Hierarchical optimized Radial Basis Function Neural Network (THORBFNN) model.

In specimen‐level avian morphology datasets, missing trait values are common, particularly for traits that are time‐consuming or difficult to measure in museum collections and field surveys. In this study, we consider the following regression problem: given a set of observed morphological and categorical predictors for each individual, we aim to learn a function that predicts the value of a continuous target trait, and then use this function to impute missing values for that trait. Figure 1 presents the overall workflow of our trait imputation method, where (a) illustrates the data cleaning process and (b) depicts the forward propagation mechanism of the proposed model architecture.

Let $D={\left\{\left(x_{i},y_{i}\right)\right\}}_{i=1}^{\mathbb{N}}$ denote the dataset for a focal trait, where $x_{i}\in R^{d}$ is the vector of predictor variables for the i‐th individual (other morphological traits and categorical attributes such as species identity and sex), and $\Gamma_{i}$ tis the original‐scale value of the target trait (e.g., wing length in mm). For numerical stability and to reduce skewness, we model the log‐transformed target:

(1) $$y_{i}=\log\left(\Gamma_{i}\right)$$

and learn a mapping *f* such that ${\hat{y}}_{i}=f\left(X_{i}\right)$ approximates $y_{i}$. Imputed values on the original scale are obtained via the inverse transform ${\hat{\Gamma }}_{i}=\exp\left({\hat{y}}_{i}\right)$.

Before fitting the model, we apply the following preprocessing steps to the raw data:

#### 3.2.1 Data Cleaning

We first remove records that fail basic quality checks or lack key fields required for modeling (e.g., quality‐control flags, essential trait values). This step yields a cleaned dataset $D_{\text{clean}}$ hat forms the basis for all subsequent analyses.

#### 3.2.2 Logarithmic Transformation of Continuous Variables

For all strictly positive continuous variables (including the target trait and continuous predictors), we apply a natural logarithm transform. This reduces right‐skew, stabilizes variance, and improves the suitability of Euclidean distances used later in the model.

#### 3.2.3 Categorical Variable Encoding

Categorical predictors such as species identity and sex are encoded as integer indices using a categorical encoding scheme (implemented via a label encoder). Each distinct category is mapped to a unique integer code. These encoded variables are then treated as numerical inputs to the model. We do not impute categorical variables in this work; they are only used as predictors.

#### 3.2.4 Feature Correlation Analysis

For each candidate predictor, we compute the Pearson correlation coefficient with the log‐transformed target y using only individuals for which both variables are observed. We use these correlations to identify traits with non‐trivial association to the target and to guide the construction of candidate feature subsets (Section 3.7).

#### 3.2.5 Train/Validation Split

The cleaned dataset is randomly partitioned into a training set (70%) and a validation set (30%). The same 70/30 split is reused consistently across all methods and feature combinations to ensure fair comparison. All model fitting and hyperparameter tuning are performed on the training set, while the validation set is used to evaluate performance and to select the best configurations.

This preprocessing results in two matrices, $X_{\text{train}}$ and $X_{\mathrm{val}}$, containing the encoded predictors, and two corresponding vectors, $y_{\text{train}}$ and $y_{\mathrm{val}}$, containing the log‐transformed target trait values.

### 3.3 Radial Basis Function Network (RBFNet)

The core prediction model in our framework is a Radial Basis Function Network (RBFNet), a single‐hidden‐layer feed‐forward neural network designed for nonlinear regression.

Let $x_{i}\in R^{d}$ denote an input vector after preprocessing. An RBFNet with k hidden units computes the output:

(2) $$\hat{y}=f\left(X\right)=\sum_{j=1}^{k}\omega_{j}\phi_{j}\left(X\right)$$

where *k* is the number of radial basis neurons, $c_{j}\in R^{d}$ is the center of the jjj‐th basis function, γ>0 is a global width (or scale) parameter shared across neurons, $\omega_{j}\in R$ is the output weight associated with the jjj‐th neuron, and $\phi_{j}\left(X\right)$ is the j‐th radial basis function, chosen here as an exponentially decaying function of the distance between X and $c_{j}$:

(3) $$\phi_{j}\left(X\right)=\exp\left(-\gamma {\left\|X-c_{j}\right\|}_{2}\right)$$

In our implementation, RBFNet is realized in PyTorch as follows:

The centers ${\left\{c_{j}\right\}}_{j=1}^{k}$ are initialized using *K*‐means clustering on the training inputs (Section 3.3).

The width parameter *γ* is treated as a hyperparameter and tuned by Bayesian optimization (Section 3.4).

The output weights $\left\{\omega_{j}\right\}$ are trainable parameters optimized by gradient‐based learning. Given a batch of inputs X and fixed centers, the network first computes the pairwise Euclidean distances between inputs and centers, then applies the radial basis function to obtain the interpolation matrix G, and finally produces predictions $\hat{y}=\mathrm{G\omega}$, where $\omega ={\left(\omega_{1},\ldots ,\omega_{k}\right)}^{T}$.

To train the network, we minimize the root mean squared error (RMSE) loss between predictions and true targets on the training set:

(4) $$L_{\text{RMSE}}=\sqrt{\frac{1}{N_{\text{train}}}\sum_{i=1}^{N_{\text{train}}}{\left(y_{i}-{\hat{y}}_{i}\right)}^{2}}$$

The loss is implemented as a differentiable layer (RMSELoss), and the parameters w are updated using the RMSprop optimizer with a fixed learning rate. Training is run for a predefined maximum number of epochs, and the configuration with the best validation performance (highest $R^{2}$, Section 3.6) is retained.

### 3.4 K‐Means Clustering for Center Initialization

Choosing appropriate centers $\left\{c_{j}\right\}$ is crucial for the effectiveness of RBF networks. Rather than treating centers as free parameters, we use *K*‐means clustering to initialize them based on the geometry of the input space.

Given the training input matrix $X_{\text{train}}$, *K*‐means with k clusters partitions the samples into k disjoint subsets and minimizes the standard within‐cluster sum‐of‐squares objective:

(5) $$J=\sum_{i=1}^{N_{\text{train}}}{\left\|X_{i}-\mu_{\mathrm{zi}}\right\|}_{2}^{2}$$

where $\mu_{1},.\ldots ,\mu_{k}$ are the cluster centroids, and $z_{i}\in \left\{1,.\ldots .,k\right\}$ indicates the cluster assignment of the i‐th training sample. We then set:

(6) $$c_{j}=\mu_{j},j=1,.\ldots ,k$$

so that each radial basis function is centered at a data‐driven prototype in the predictor space. This initialization aligns the RBFNet's receptive fields with regions of high data density and has been found to improve both convergence and predictive performance compared to random initialization.

In our experiments, the number of clusters *k* is treated as a hyperparameter and is optimized using Bayesian search (Section 3.4) within a predefined range.

### 3.5 Hierarchical Optimization Strategy

The RBFNet described above depends on two key hyperparameters:

- The number of radial basis neurons *k*, which also equals the number of *K*‐means clusters, and
- The width parameter *γ*, which controls the smoothness of the radial basis functions.

We define the hyperparameter space as:

- $k\in \left[10,25\right]$ (integer),
- $\gamma \in \left[0.05,0.5\right]$ (continuous).

For a given feature combination (Section 3.7), each evaluation of the objective function proceeds as follows:

1. Initialize an RBFNet with the proposed hyperparameters k and γ.
2. Run K‐means with k clusters on $X_{\text{train}}$ to obtain centers $\left\{c_{j}\right\}$.
3. Train the RBFNet on $X_{\text{train}}$ and $y_{\text{train}}$ using RMSE loss and RMSprop.

Compute predictions $y_{\mathrm{val}}$ on the validation set and calculate the coefficient of determination $R^{2}$.

The objective to be minimized by the optimizer is the negative validation $R^{2}$:

(7) $$J\left(k,\gamma \right)=-R_{\mathrm{val}}^{20}\left(k,\gamma \right)$$

so that lower values of J correspond to better models. We perform Bayesian optimization using a Gaussian‐process surrogate model with an acquisition function based on expected improvement, and run a fixed number of calls (10 evaluations per feature combination). The best pair (k*, γ*) is selected as the one achieving the highest validation $R^{2}$ (Algorithm 1).

### 3.6 Overall Training Pipeline

ALGORITHM 1 **Training Procedure of THORBFNN.**

**Input:** Dataset $D={\left\{\left(x_{i},y_{i}\right)\right\}}_{i=1}^{N}$,    Feature set $x_{i}\in R^{d}$,    Hyperparameters: *k*, *γ*, learning rate *η***Output:** Trained RBF model *f*(x)**Step 1: Feature Selection and Transformation**Apply log transformation to *y*_*i*_Apply label encoding/log transformation to x_*i*_**Step 2: Dataset Splitting**Randomly split D into $D_{\text{train}}$ and $D_{\mathrm{val}}$**Step 3: Clustering Initialization**Apply KMeans on $D_{\text{train}}$ to obtain centers ${\left\{c_{j}\right\}}_{j=1}^{k}$**Step 4: Radial Basis Calculation**Compute:    $\phi_{j}\left(x_{i}\right)=\exp\left(-\gamma {\left\|x_{i}-c_{j}\right\|}^{2}\right)$Construct interpolation matrix $G\in R^{N\times k}$**Step 5: Model Training via Backpropagation**Initialize weights ${\left\{w_{j}\right\}}_{j=1}^{k}$ randomly**for** *t* = 1 *to max_epoch* **do**    Compute prediction: ${\hat{y}}_{i}=\sum_{j=1}^{k}w_{j}\cdot \phi_{j}\left(x_{i}\right)$    Compute loss:    $L=\sqrt{\frac{1}{N}\sum_{i=1}^{N}{\left(y_{i}-{\hat{y}}_{i}\right)}^{2}}$    Update weights: $w_{j}\leftarrow w_{j}-\eta \cdot \frac{\partial L}{\partial w_{j}}$**endStep 6: Hyperparameter Optimizationfor** *each feature subset* **do**    **for** (*k*, *γ*)~*Bayesian search* **do**    Train model as above and record validation *R*^2^    **end**    Retain best (k*, γ*) and corresponding model**endreturn** final model $f\left(x\right)=\sum_{j=1}^{k^{*}}w_{j}\cdot \phi_{j}\left(x\right)$

### 3.7 Evaluation Metrics

To comprehensively evaluate the performance of the proposed model, the following metrics were adopted:

These metrics provide a multifaceted view of model performance, enabling targeted improvements and comparisons.

### 3.8 Feature Combination Search and Result Saving

To explore which subsets of predictors contribute most to accurate trait imputation, we conduct a structured feature combination search.

#### 3.8.1 Generation of Candidate Feature Combinations

For each target trait, we start from a predefined list of candidate predictors (e.g., selected morphological measurements or sensor signals). We then generate all combinations of these predictors up to a maximum size of three variables. Combinations of size one are excluded because preliminary experiments showed they are generally insufficient to capture the multivariate structure of trait relationships.

#### 3.8.2 Training and Validation for Each Combination

For every feature combination, we preprocess the corresponding columns using the scheme described in Section 3.1: run Bayesian hyperparameter optimization to select the best (k, γ); train the RBFNet with the best hyperparameters; compute validation metrics (Section 3.6) and store the validation predictions and true values.

#### 3.8.3 Ranking and Selection of Top Combinations

We rank feature combinations by their best validation $R^{2}$. For each target trait, we retain the top five combinations as the most informative subsets.

#### 3.8.4 Saving Results

For each of these top combinations, we export an Excel file containing the true trait values and the corresponding predictions on the original scale (obtained by exponentiating log‐scale predictions). These files facilitate detailed downstream analysis, such as inspecting residual distributions or comparing imputation quality across traits and feature sets.

This nested feature‐combination and hyperparameter search provides a principled way to identify compact yet informative predictor sets for each target trait, while also supplying transparent result files for further ecological interpretation.

## 4 Experiment

### 4.1 Experimental Configuration

Datasets were obtained from AVONET Data S1. Then we conducted all experiments on an NVIDIA RTX 3080 processor with 24GB of GPU memory. We built the model using PyTorch as the deep learning framework, and we used Linux as the operating system, and we fixed the random seeds throughout the training.

All experiments are implemented in Python using PyTorch for the RBFNet model and scikit‐learn for preprocessing and baseline utilities. Bayesian hyperparameter optimization is carried out using a Gaussian‐process‐based optimizer.

To ensure reproducibility, we fix random seeds for the Python random module, NumPy, and PyTorch (including CUDA backends) and disable nondeterministic cuDNN optimizations. All results reported in this paper are based on the same 70/30 train/validation split for each target trait and the same sequence of random seeds for hyperparameter search.

We use a fixed maximum number of training epochs and the same optimizer settings (RMSprop learning rate) across all experiments, so that performance differences primarily reflect model design and hyperparameter choices rather than arbitrary training schedules.

### 4.2 Results

#### 4.2.1 Ablation Experiments

To further validate the effectiveness of our model in capturing meaningful feature dependencies, we conduct an ablation study comparing performance under two distinct training scenarios: one using only complete (i.e., non‐missing) data, and another using data where missing values are imputed via mean substitution.

In the first scenario, we construct a baseline training set by removing all instances that contain any missing values. This clean data set is randomly divided into 70% for training and 30% for testing. To ensure consistent evaluation across conditions, we retain the same subset of tests 30% in all experimental settings.

In the second scenario, we start with the original datasets containing missing values. Missing entries are imputed using feature‐wise mean values computed from the complete cases. To avoid data leakage, we exclude the previously selected test instances and use the remaining data (approximately 70%) as the imputed training set.

We then train two separate models, one on the clean training set and the other on the mean input training set, and evaluate both on the shared test set using the metrics defined in Table 1. A significant drop in performance when training on the imputed datasets would indicate that our model is leveraging informative patterns in the true data distribution, and that imputation introduces noise that hinders generalization.

**TABLE 1:** Evaluation Metrics.

Metric	Formula	Description
MSE (Mean Squared Error)	$\frac{1}{N}\sum {\left(y-\hat{y}\right)}^{2}$	Average squared error
RMSE (Root Mean Squared Error)	$\sqrt{\mathrm{MSE}}$	Standard deviation of prediction errors
MAE (Mean Absolute Error)	$\frac{1}{N}\sum \left|y-\hat{y}\right|$	Robust error metric
MedAE (Median Absolute Error)	$\text{median}\left(\left|y-\hat{y}\right|\right)$	Resilient to outliers
MAPE (Mean Absolute Percentage Error)	$\frac{1}{N}\sum \left|\frac{y-\hat{y}}{y}\right|$	Relative error measure
MSLE (Mean Squared Logarithmic Error)	$\frac{1}{N}\sum {\left(\log\left(1+y\right)-\log\left(1+\hat{y}\right)\right)}^{2}$	Emphasizes small‐value predictions
*R* ^2^ (Coefficient of Determination)	$1-\frac{\sum {\left(y-\hat{y}\right)}^{2}}{\sum {\left(y-\bar{y}\right)}^{2}}$	Goodness‐of‐fit indicator

This ablation study offers insight into whether the model benefits from learning genuine feature interactions rather than overfitting to statistical artifacts introduced during imputation.

Due to the limited space of the article, we only provide some of the most prominent feature comparisons in the text. However, in Figures 4 and 5, we will show the differences between the interpolated values and the true values of more traits in the test set.

**FIGURE 2:**
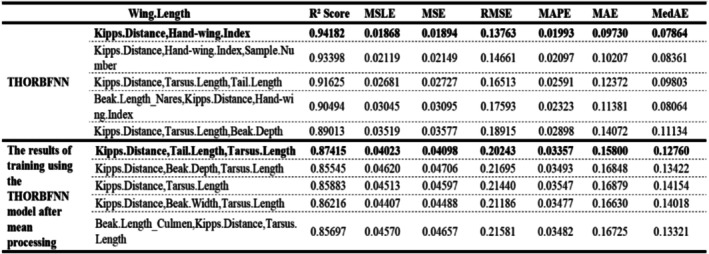
The figure shows the fitting results of the Wing. Length on our model after supplementing the original data with the original value and the average value respectively. Among them, bold indicates the best restoration effect.

**FIGURE 3:**
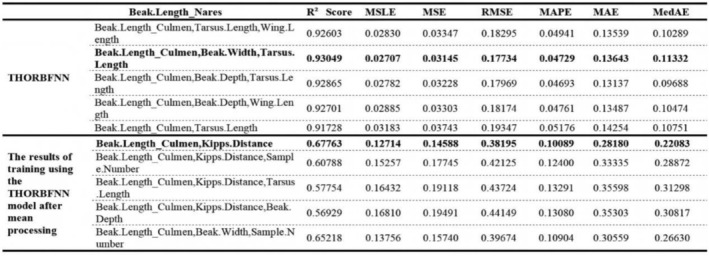
The figure shows the fitting results of the distance length from the beak to the nostrils of birds on our model after supplementing the original data with the original values and the average value respectively. Among them, bold indicates that the reduction effect is the best.

**FIGURE 4:**
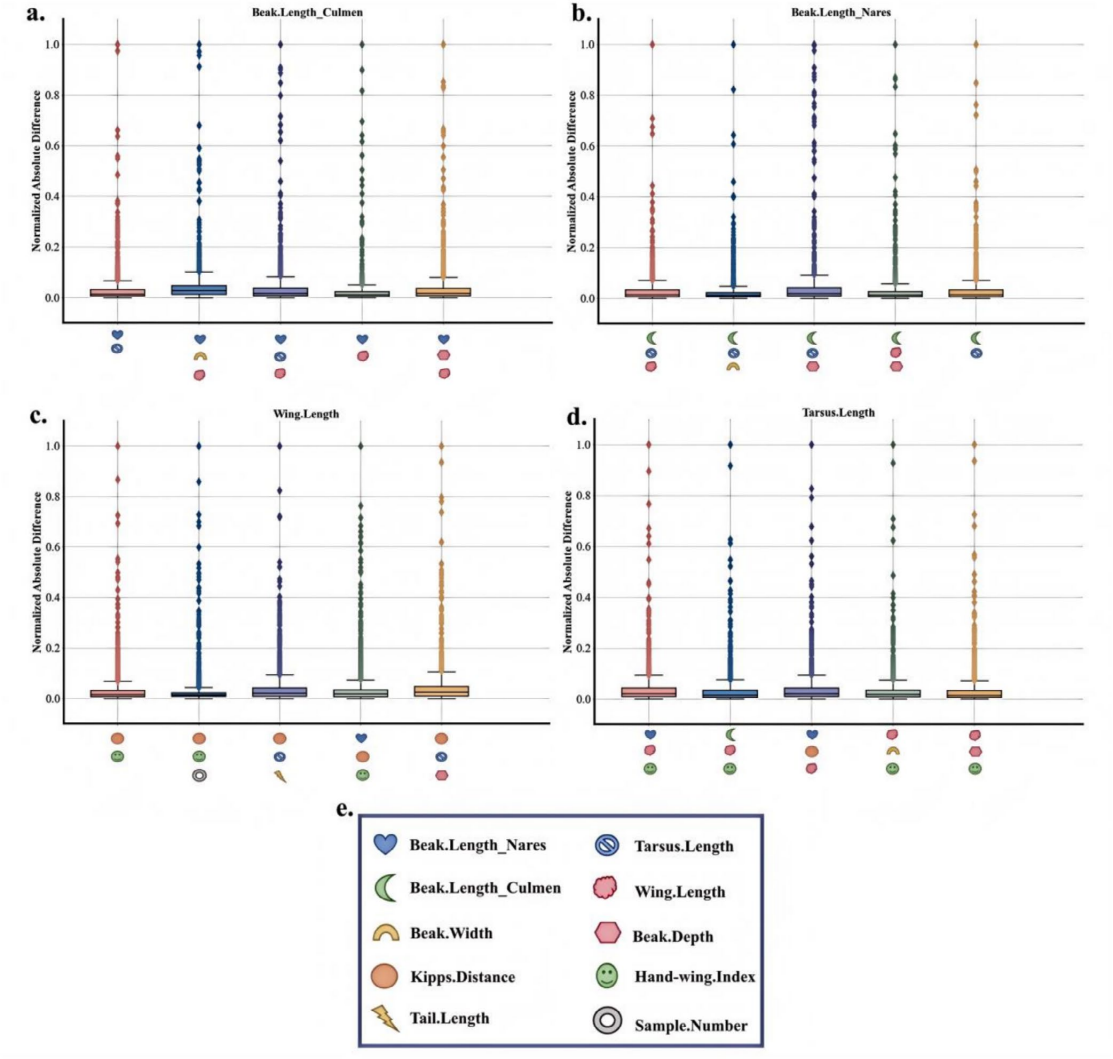
The figure shows the visualization of the gap between the predicted values and the true values of our model in the trait interpolation tasks of Beak.Length Nares, Beak.Length Culmen, Tarsus. Length and Wing.Length. Among them, the abscesses of (a–d) represent different trait combinations. The vertical coordinate represents the gap between the predicted value and the true value, and (e) represents the traits represented by different trait identifiers.

**FIGURE 5:**
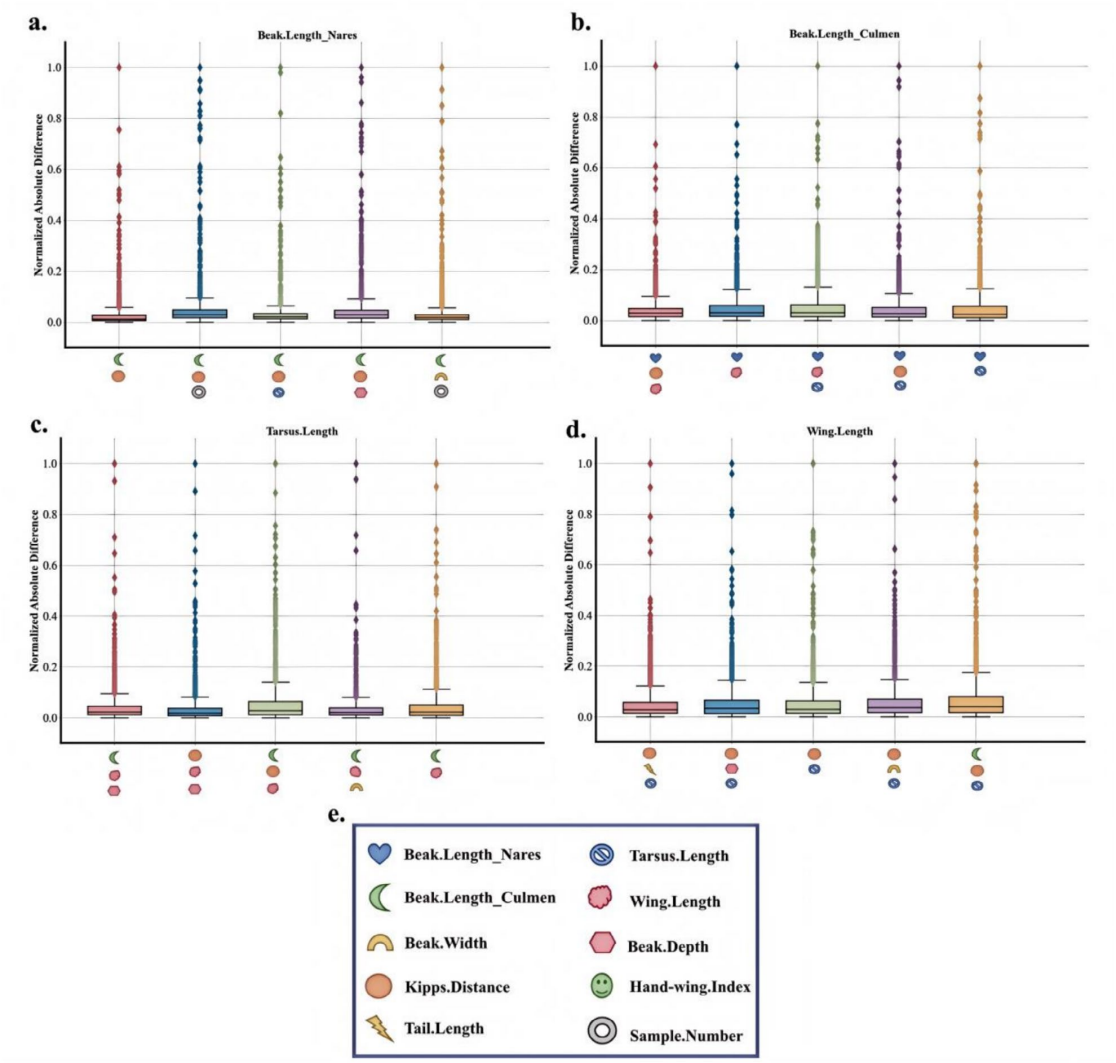
The figure shows the visualization of the gap between the predicted values and the true values of the interpolation tasks for the traits of Beak.Length Nares, Beak.Length Culmen, Tarsus.Length and Wing.Length on our model after using mean interpolation to interpolate the missing values of the original data. Among them, The abscissa of (a–d) represents different combinations of traits, the ordinate represents the gap between the predicted values and the true values, and (e) represents the traits represented by different trait identifiers.

It can be seen from Figures 2, 3, 4, 5 that when the data after mean imputation is put into model training, it will have an impact on the trend of the model's learning features. From the correspondence on the abscissa of each subgraph, it can be seen that in the case of direct imputation using the original data, the effects of different features on the target interpolated features are different, and the features interpolated using the original data generally have a smaller gap between the predicted values and the true values in the test set. Thus, it can be concluded that our model can better capture the connections among various features. It can provide an effective method for supplementing missing data.

**FIGURE 6:**
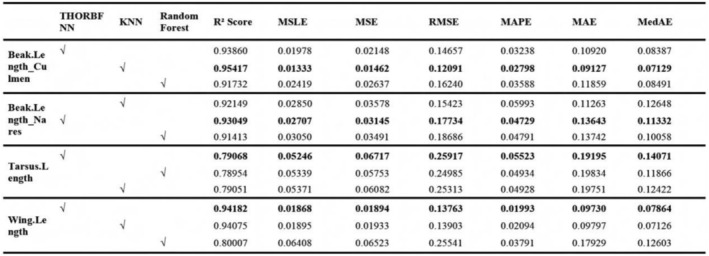
The figure shows the comparison results with the KNN and Random Forest models, with a total of four traits. Among them, the bold type represents the model with the best effect. The selected areas are the models used for each trait.

**FIGURE 7:**
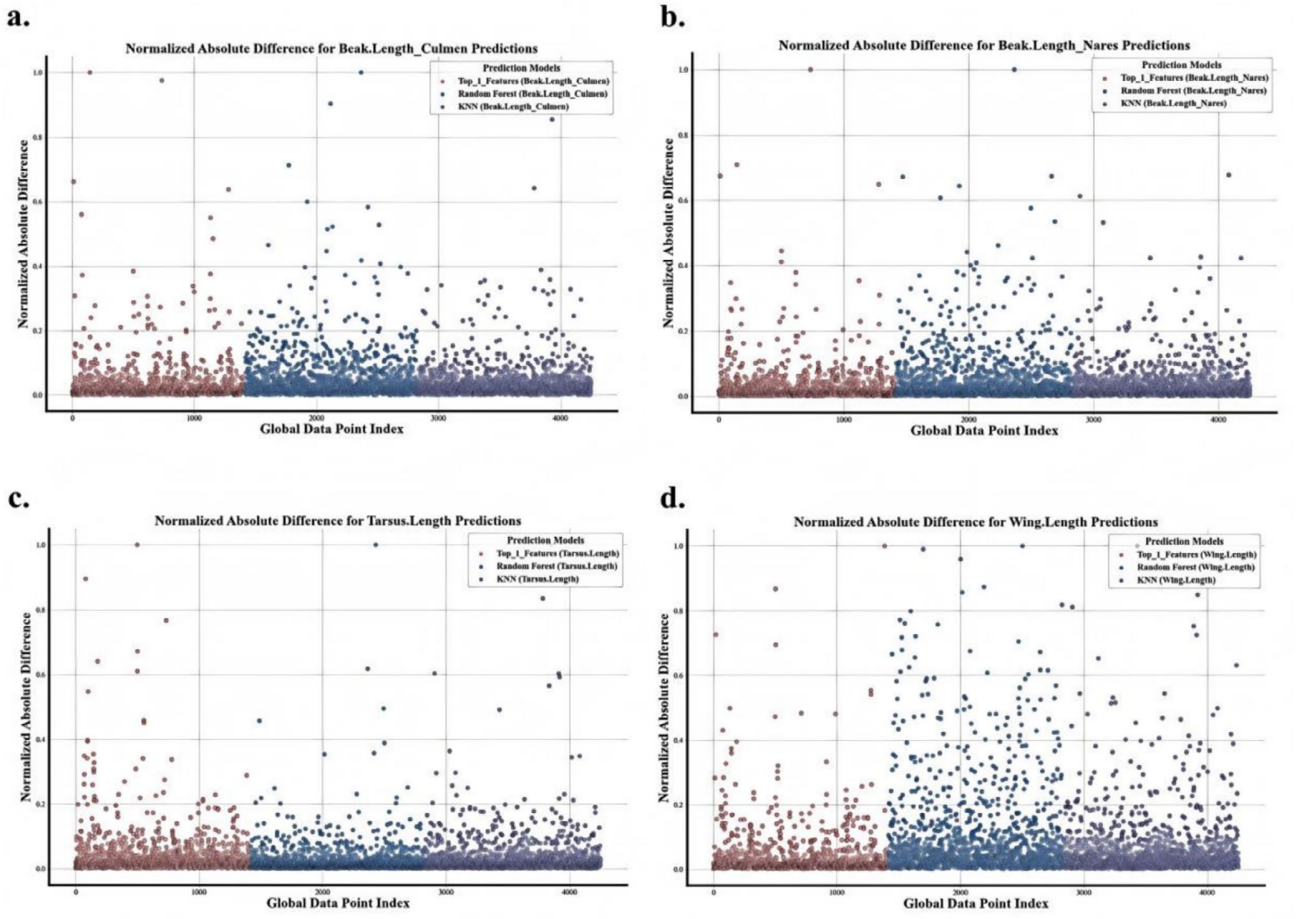
The figure shows the comparison of imputation performance across four traits using THORBFNN, KNN, and Random Forest. (a) Beak.Length_Culmen, (b) Beak.Length_Nares, (c) Tarsus.Length, (d) Wing.Length. In each subplot, different colors represent different models: blue for THORBFNN, orange for KNN, and green for Random Forest.

#### 4.2.2 Comparative Experiment

In the comparative experiments, we evaluated our proposed THORBFNN model against two widely used imputation methods: *k*‐nearest neighbors (KNN) and random forest.

After preprocessing and cleaning the datasets, we randomly split it into 70% for training and 30% for testing. Each of the three models: KNN, random forest, and THORBFNN was trained on the training set and evaluated on the test set using the metrics defined in Table 1.

To ensure a fair and credible comparison, we used the same set of input features for all methods, corresponding to the optimal trait combinations identified by the THORBFNN model for each target trait. The specific experimental results are shown in Figures 6 and 7.

It can be seen from Figures 6 and 7 that our model is superior to the KNN and random forest models in most cases. Especially from the scatter plot of the gap between the predicted values and the true values in Figure 7, it can be observed that when our model performs imputation for different traits, most of the differences tend to zero, while the differences of the other two models show a more discrete outward expansion phenomenon. Once again, it has been proven that our model can learn the correlation between features well, thereby completing the data imputation task very well.

## 5 Discussion

This study introduces THORBFNN, a hybrid imputation framework designed for avian morphological trait datasets with heterogeneous missingness. The empirical results indicate that THORBFNN achieves consistently better overall imputation quality than widely used baselines, supporting the view that combining structure‐aware partitioning with nonlinear function approximation is advantageous for trait recovery in large, noisy ecological datasets.

### 5.1 Why THORBFNN Improves Trait Recovery

A central methodological contribution is the explicit separation of global structure discovery and local nonlinear mapping. Regularized K‐means partitions individuals into morphologically coherent groups, which helps preserve local trait structure and mitigates the risk that a single global model averages across ecologically distinct regimes. Within each cluster, the RBFNN provides flexible nonlinear approximation, allowing the model to learn trait covariation patterns that are difficult to capture with distance‐based or tree‐based imputers when missingness is substantial. The hierarchical Bayesian optimization further stabilizes performance by tuning cluster granularity and kernel width in a data‐driven manner rather than relying on ad hoc defaults.

### 5.2 Relation to Existing Machine‐Learning Applications in Trait‐Based Ecology

Recent studies increasingly apply machine learning to connect morphology with ecological function and performance. For instance, morphological traits have been used to predict avian primary lifestyle using extreme gradient boosting, which highlights the predictive utility of multivariate trait combinations for ecological categorization (Fakhrzad et al. 2025). In addition, morphology‐based prediction of multivariate performance phenotypes has been demonstrated in lizards, indicating that nonlinear multivariate relationships between morphology and function can be learned with high fidelity (Lailvaux et al. 2022). Beyond zoological applications, machine learning has also been adopted to predict agronomic traits and to support optimization decisions, further demonstrating the broader value of data‐driven nonlinear models for trait prediction and decision support (Egwumah et al. 2025). In avian ecology, machine‐learning models have been used to predict bird species detection from environmental factors and tree characteristics, which reflects the growing acceptance of machine‐learning frameworks for complex ecological inference (Madrigal‐Roca 2024).

In contrast to the above studies, which primarily focus on prediction or classification tasks, the present work addresses a complementary and foundational problem, namely the recovery of missing trait measurements that is required for downstream ecological and evolutionary analyses. By improving imputation fidelity without relying on phylogenetic inputs, THORBFNN can serve as a practical preprocessing component for large trait databases in settings where phylogenies are unavailable, uncertain, or difficult to integrate at scale.

### 5.3 Ecological Implications and Robustness Considerations

Accurate imputation is not only a numerical objective but also a prerequisite for reliable inference of trait covariation, functional diversity, and trait–environment relationships. The ablation comparison against mean‐imputed training indicates that THORBFNN captures meaningful covariation rather than artifacts induced by simplistic filling. Nevertheless, the validity of any imputation method depends on the missing‐data mechanism. If missingness is systematically associated with geography, taxonomy, or measurement protocols, additional sensitivity analyses (e.g., stratified evaluation by region or clade, or controlled missingness simulations) are warranted to quantify potential biases.

### 5.4 Limitations and Future Directions

First, the current framework uses correlation‐based feature selection, which is computationally efficient but may overlook informative predictors with weak marginal correlation yet strong conditional influence; future extensions could consider regularized multivariate screening or model‐based selection. Second, evaluation is conducted on a fixed split; repeated splits or nested evaluation would provide a more conservative estimate of generalization. Third, while THORBFNN does not require phylogenetic information, integrating phylogenetic structure or hierarchical random effects may further improve imputations when missingness aligns with evolutionary relatedness. Finally, extending the framework to semi‐supervised settings and uncertainty‐aware imputation could better support downstream analyses that propagate imputation uncertainty.

## 6 Conclusion

This study presents THORBFNN, a hybrid imputation framework for avian morphological trait datasets with pervasive missing values. THORBFNN integrates regularized clustering to preserve local morphological structure, cluster‐wise RBF‐based nonlinear modeling to capture trait relationships within coherent groups, and hierarchical Bayesian optimization to tune key hyperparameters under heterogeneous missingness. Experiments on a global AVONET‐based dataset show that THORBFNN achieves more accurate and stable trait recovery than standard machine‐learning baselines. Ablation analyses further suggest that the performance improvements arise from learning meaningful trait covariation rather than relying on artifacts introduced by simplistic imputation strategies. Collectively, these results indicate that THORBFNN is a practical and scalable approach for completing large trait databases without requiring phylogenetic information, thereby supporting downstream biodiversity analyses that depend on robust multivariate trait profiles. Future work will evaluate robustness across missingness mechanisms and taxa, and extend the framework toward uncertainty‐aware and semi‐supervised imputation.

## Author Contributions

**Yu Bai:** conceptualization (equal), formal analysis (equal), writing – original draft (equal). **Pengfei Song:** funding acquisition (equal), resources (equal). **Shimin Wen:** software (equal), validation (equal). **Hongjie Zhu:** data curation (equal), investigation (equal), methodology (equal). **Yuang Wang:** visualization (equal), writing – original draft (equal). **Huazhang Wang:** resources (equal), validation (equal), writing – review and editing (equal). **Xixi Feng:** funding acquisition (equal), project administration (equal). **Bo Ma:** project administration (equal), validation (equal), writing – review and editing (equal). **Daji Ergu:** project administration (equal), writing – review and editing (equal). **Fangyao Liu:** conceptualization (equal), funding acquisition (equal), project administration (equal), resources (equal), writing – review and editing (equal).

## Funding

This project is supported by the 2023 award fund of Qinghai Provincial Key Laboratory of Animal Ecological Genomics. This work is also supported by the Fundamental Research Funds for the Central Universities, Southwest Minzu University (ZYN2026069). This project is also supported by the National Natural Science Foundation of China under Grant (82474353, 72174172).

## Conflicts of Interest

The authors declare no conflicts of interest.

## Supporting information


**Data S1:** ece373173‐sup‐0001‐supinfo.xlsx.

## Data Availability

Data is available in this public platform: https://figshare.com/s/b990722d72a26b5bfead. In accordance with the requirements, we have strictly adhered to reference standards for data citation in the paper.
